# Natural Conception May Be an Acceptable Option in HIV-Serodiscordant Couples in Resource Limited Settings

**DOI:** 10.1371/journal.pone.0142085

**Published:** 2015-11-05

**Authors:** Lijun Sun, Fang Wang, An Liu, Ruolei Xin, Yunxia Zhu, Jianwei Li, Ying Shao, Jiangzhu Ye, Danqing Chen, Zaicun Li

**Affiliations:** 1 Center for Infectious Diseases, Beijing Youan Hospital, Capital Medical University, Beijing, 100069, China; 2 National Center for Women and Children’s Health, China CDC, Beijing, 100081, China; 3 Beijing Center for Preventive Medicine Research, Beijing, 100013, China; 4 Department of Gynecology, Beijing Youan Hospital, Capital Medical University, Beijing, 100069, China; 5 Women’s Hospital, School of Medicine, Zhejiang University, Hangzhou, 310006, Zhejiang Province, China; University of Athens, Medical School, GREECE

## Abstract

Many HIV serodiscordant couples have a strong desire to have their own biological children. Natural conception may be the only choice in some resource limited settings but data about natural conception is limited. Here, we reported our findings of natural conception in HIV serodiscordant couples. Between January 2008 and June 2014, we retrospectively collected data on 91 HIV serodiscordant couples presenting to Beijing Youan Hospital with childbearing desires. HIV counseling, effective ART on HIV infected partners, pre-exposure prophylaxis (PrEP) and post-exposure prophylaxis (PEP) in negative female partners and timed intercourse were used to maximally reduce the risk of HIV transmission. Of the 91 HIV serodiscordant couples, 43 were positive in male partners and 48 were positive in female partners. There were 196 unprotected vaginal intercourses, 100 natural conception and 97 newborns. There were no cases of HIV seroconversion in uninfected sexual partners. Natural conception may be an acceptable option in HIV-serodiscordant couples in resource limited settings if HIV-positive individuals have undetectable viremia on HAART, combined with HIV counseling, PrEP, PEP and timed intercourse.

## Introduction

HIV serodiscordant couples represent at least half of all HIV-affected couples worldwide. Many of these couples have childbearing desires. It is reported that 25–63% of HIV-infected men and women desire children [1–3]. With HIV serodiscordance (when one person is infected and the other is not), it is important for couples and health care providers to carefully consider and optimize the balance between attempts to conceive and risk of HIV transmission [4]. Because HIV is an incurable sexually transmitted infection, unprotected sex between serodiscordant couples is highly discouraged. Although condom use may prevent horizontal transmission, it also prevents pregnancy. Prior to the widespread availability of antiretroviral therapy (ART), it was almost impossible for a HIV-infected man to have his own child safely and the desire to conceive among HIV-infected women were discouraged due to a high perinatal transmission risk [5].

A series of study suggested that in clinical trial settings, heterosexual HIV transmission can be reduced through regular viral load testing, ART and sustained viral suppression, but such testing and treatments may not be widely accessible due to structural or individual-level barriers, even in resource-rich countries [6,7]. Given the increasing fertility desires among HIV-affected couples, other strategies to reduce heterosexual viral transmission such as timed condom less sex are essential. Washed intrauterine insemination (IUI) and in-vitro fertilization (IVF) are good options for HIV-serodiscordant couples, which can eliminate the need for intercourse and subsequent risk of HIV transmission. Unfortunately, the techniques may be not available in many countries or areas and the cost of assisted reproductive services can be prohibitive for many people [8]. In addition, the pregnancy rate per cycle of IUI is only 10% to 15% [9,10]. It may take several cycles for a woman to get pregnant.

The introduction of ART has not only improved life expectancy dramatically, but also significantly reduced the risk of sexual transmission of HIV between serodicordant couples [11,12]. In a prospective cohort analysis, a 92% reduction in HIV transmission risk was found following ART among HIV serodiscordant couples [13]. In a sensitivity analysis including all 6 studies, the rate of transmission was 0 per 100 person-years (95%CI  =  0–0.01) after omitting all transmissions with known detectable or unconfirmed viral loads [14]. A systematic review also confirmed that ART can effectively avoid horizontal and vertical transmission in HIV serodiscordant couples [15]. Effective antiviral treatment provides a basis for safe natural conception. As indicated by one Spanish study, natural conception could be considered a possible alternative for HIV-serodiscordant couples, as long as complete suppression of viremia with HAART is achieved in the infected partner [16]. To further reduce the transmission risk, timed intercourse with PrEP was used, and achieved a high pregnancy rate of 75% [17].

However, little is known about natural pregnancy in HIV discordant couples in resource limited settings. Here, we reported a retrospective study concerning HIV discordant couples who attained natural pregnancies in China.

## Method

### Study population

A retrospective study was conducted in HIV-1 serodiscordant couples who desired to conceive a child and followed up in STD/AIDS clinic of Beijing Youan Hospital, Capital Medical University, from January 2008 and June 2014. Basic HIV knowledge and risk of infection during natural conception were told to all couples.

### Preparation for fertilization

Pre-pregnancy examination was performed in all female partners, including infection screening and gynecologic examination. Liver and renal function was also tested for the feasibility of ART. Folic acid was taken for at least 3 months. To determine the optimal time of conception (36 h after LH-peak), according to the convenience of the participants and the availability of the test, LH in the urine was tested by urine LH Ovulation Test Kit (Gemc Technology Co., Ltd, Zhengzhou, China), combined with basal body temperature or ovary Ultrasound in some females (females who were not suitable for basal body temperature and whose test strip assay for ovulation were not ideal) to monitor the ovulation. Of them, some worked on night shift, thus basal body temperature in the morning cannot be guaranteed; some lived in remote regions where test strips could not be easily acquired. Additionally, for the females who did not conceive within three menstrual cycles, some of them received poor test strip assay results of continuous tests.

In serodiscordant couples with male positive partner, HIV infected male partner was treated with ART for at least 6 months to keep HIV-RNA undetectable in plasma (<400 copies/ml). Active genital infections were excluded. Semen examination was performed. Sexual life was prohibited before one week of scheduled conception. In serodiscordant couples with female positive partner, semen was injected into vagina with a syringe by female positive partners or their husbands at home, which had no risk of HIV infection for the male partner. If the first attempt was unsuccessful, a second one would begin without PrEP interval.

### Prevention of HIV infection

In the couples with female positive partners, if the infected mothers were not treated yet, they would take ART at 14 weeks of pregnancy for prevention of mother to child transmission (PMTCT). If EFV containing regimens were taken by the infected mothers, EFV was switched to Lopinavir/Ritonaivr at least 6 months before conception. While in the couples with male positive partners, timed intercourse with PrEP and PEP were used to reduce the risk of HIV infection. AZT or TDF+3TC+ LPV/r was taken by the seronegative female partner from the morning of LH-peak till 28 days after the last unprotected sex in one cycle. All babies born to HIV-infected mothers received prophylaxis with zidovudine after birth.

The possibility of vertical HIV transmission was determined according to HIV antibody screening and confirmatory tests and polymerase chain reaction (PCR) at 1, 3, 6, and 12 months of age in the baby. The test of HIV infection was performed on seronegative female partners at 6th, 12th and 24th week after the last unprotected intercourse.

### Ethical considerations

Ethical approval was obtained from the Ethics Committee of Beijing Youan Hospital, Capital Medical University. All participants signed an informed consent.

## Results

A total of 91 HIV-serodiscordant couples were enrolled in the study, among which 44 were positive in male partners and 47 were positive in female partners. At baseline, mean age was 30.6±3.9 years for the HIV positive male partners mainly infected by homosexual behavior (Table 1), and 27.3±4.2 years for the HIV positive female partners mainly infected by heterosexual behavior (Table 2). All infected male partner had received ART for more than 6 months and achieved undetectable plasma HIV RNA before timed intercourse.

**Table 1:** The baseline characteristics of HIV positive men with female HIV negative partners.

items	value (N = 44)
age (mean±sd, years)	30.6±3.9
transmission route (N, %)	
homosexual	41 (93.2)
heterosexual	2 (4.5)
blood	1 (2.3)
baseline plasma HIV viral load (median, range, copies/ml)	5873 (<50, 100000)
<50	2
50–9999	32
10000–49999	8
>50000	2
baseline CD4+ T cell count (median, range, cells/ml)	257.6 (16.6, 746.0)
<200	19
200–349	12
350–499	9
>500	4
ART regimen (n)	
AZT(or TDF) +3TC+EFV	32
AZT+3TC+NVP	3
AZT(or TDF) +3TC+LPV/r	8
untreated	1
duration of ART (median, range, years)	3.0 (1.0, 8.0)
plasma HIV viral load at conceive (copies/ml)	
<50	42
50–100	2
CD4+ T cell count at conceive (median, range, cells/ml)	480.0 (298.0, 855.0)
<200	2
200–349	5
350–499	21
>500	16
ART regimen for female partner after conceive	
AZT+3TC+LPV/r	31
TDF+3TC+LPV/r	3
AZT	1
untreated	9
duration of ART for female partner after conceive (median, range, days)	67 (0, 175)

**Table 2:** The baseline characteristics of HIV positive women with male HIV negative partners.

items	value (N = 47)
age (mean±sd, years)	27.3±4.2
transmission route (n, %)	
homosexual	0 (0)
heterosexual	24 (51.1)
blood	23 (48.9)
baseline plasma HIV viral load (median, range, copies/ml)	5000 (<50, 91000)
<50	10
50–9999	19
10000–49999	9
>50000	3
missing data	6
baseline CD4+ T cell count (median, range, cells/ml)	318.7 (68.9, 806.0)
<200	10
200–349	12
350–499	10
>500	10
missing data	5
ART regimen during pregnant(n)	
AZT(or D4T) +3TC+EFV	4
AZT+3TC+NVP	13
AZT(or TDF) +3TC+LPV/r	30
ART start gestational week (median, range)	24 (0, 43)
duration of ART (median, range, days)	112 (0, 280)
plasma HIV viral load at delivery (copies/ml)	
<50	4
50–400	32
>400	2
missing data	9
CD4+ T cell count at delivery (median, range, cells/ml)	440.1 (134.5, 787.0)
<200	2
200–349	10
350–499	14
>500	16
missing data	5
ART regimen for newborns	
AZT	7
NVP	17
AZT+NVP	19
TDF +3TC+LPV/r	1

During the observation time, 44 male positive couples had a total number of 169 documented unprotected events of vaginal intercourse (Table 3). None of the female partners had seroconverted for HIV. There were 35 times of successful conception in the male positive couples, and 36 newborns living with HIV antibody negative. Of the 30 HIV infected male partners taking an EFV based regimen at baseline, no one changed EFV to other drugs. However, no malformation was found in their newborns.

**Table 3:** Natural pregnancy outcomes of HIV-discordant couples.

items	male+, female- (n = 44)	male-, female+ (n = 47)
Event of unprotected intercourse	169	no
Natural conception	35	47
termination of pregnancy	-	2
Fetus death due to umbilical cord around neck	-	1
newborn	36	44

Since semen was injected by syringe, there was no unprotected vaginal intercourse in female positive serodiscordant couples. Viral load was undetectable all the infected mothers at the time of delivery. Among the 47 successful conceptions in the female positive couples, 2 were miscarriage due to unknown reasons, 44 were living newborn without HIV infection and 1 stillbirth due to umbilical cord around the neck in late pregnancy.

## Discussion

Intrauterine insemination (IUI) and in vitro fertilization (IVF) for HIV-infected people are still not available in China by now. Reproductive options are limited in developing countries such as China. With the development of ART in treating HIV infected patients and preventing transmission, natural conception may be a relatively safe and exciting option for many HIV-discordant couples to have their own biological children. Our results showed that there were no cases of horizontal seroconversion in couples of HIV positive partners receiving suppressive HAART, which were consistent with previous reports [18,19].

The first data on natural conception in HIV-discordant couples[20], published in the Lancet in 1997, found that 4% of the women became HIV-positive some time after a natural pregnancy, but not all of their partners were on antiretroviral therapy. In 2006, A Spanish study [19] reported 76 natural conceptions of 62 HIV-discordant couples in which the HIV-infected partner is receiving maximally suppressive HAART. None of the HIV-negative partners became HIV-infected during natural conception. In our study, all the HIV-discordant couples received HAART to keep a sustained viral load suppression and their CD4 cell counts were at an acceptable level at the time of delivery, which may contribute to the successful reproductive outcome.

The most concern issue in natural conception is risk of HIV transmission. Based on a large retrospective observational cohort study in China, the rate of HIV infection was 0.8 per 100 person-years among HIV discordant couples on ART, which had a 66% relative reduction in HIV incidence compared with non-treatment group [21]. In this study, a combination of HIV counseling, STI screening, effective ART on HIV infected partners, PrEP and PEP in negative partners and timed intercoursemaximally reduces risk of HIV transmission. A higher pregnancy rate was also observed, which could be due to the unprotected intercourses at ovulation period. Although there are no horizontal transmissions in our study, it does not mean that there is no risk of HIV transmission in natural conception. Risks and benefits should be weighed when choosing natural conception.

Because of potential teratogenicity of EFV, it is not recommended to be used in women who wish conceive or are sexually active and not using effective contraception [22]. For pregnant women, EFV treatment is recommended to be started after the 8th week of pregnancy [23]. However, if the male infected partners had already taken EFV containing regimen in our study, the regimen was continued without change due to the limited choice of ART and no data so far to indicate the potential harm to the fetus if the father is taking EFV. Malformation was not found in their newborns.

This study also has some limitations, first, only a small number of HIV-serodiscordant couples were included in this study, which limited the statistic power of this study. Second, due to the sustained viral suppression, there is no horizontal transmission in our study, therefore, in this study, no analytic results for factors affecting infection rate during natural conception could be drawn. A further large study should be conducted to achieve more detailed results.

In summary, natural conception may be an acceptable option in HIV-serodiscordant couples in resource limited settings if HIV-positive individuals have undetectable viremia on HAART, combined with HIV counseling, PrEP, PEP and timed intercourse. Regular detection of HIV, ART and sustained viral suppression can greatly reduce the risk of HIV transmission during natural conception. Natural conception should be only recommended if HIV is well controlled by ART in the positive partner.
